# Crystal Structures of *Arabidopsis thaliana* GDP-D-Mannose Pyrophosphorylase VITAMIN C DEFECTIVE 1

**DOI:** 10.3389/fpls.2022.899738

**Published:** 2022-05-23

**Authors:** Chi Zhang, Shun Zhao, Yu-Shuai Li, Chao He, Xiao Wang, Lin Liu

**Affiliations:** ^1^School of Life Sciences, Anhui University, Hefei, China; ^2^Key Laboratory of Photobiology, Institute of Botany, Chinese Academy of Sciences, Beijing, China; ^3^Anhui Key Laboratory of Modern Biomanufacturing, Anhui University, Hefei, China

**Keywords:** ascorbic acid, nucleotide sugar, guanylyltransferase, crystallography, oligomerization

## Abstract

Plant GDP-D-mannose pyrophosphorylase (GMPase) catalyzes a committed step in ascorbic acid biosynthesis pathway. *Arabidopsis thaliana* VTC1 is the first genetically characterized plant GMPase and has unique properties when compared with bacterial and animal homologs. Here we present the crystal structures of VTC1 in the unliganded and product-bound states at resolutions of 2.8 and 3.0 Å, respectively. VTC1 dimerizes in a same way like other known GMPases, but dodecamerizes in a previously unobserved arrangement. The interactions to GDP-D-mannose and inorganic pyrophosphate are revealed by the product-bound VTC1 structure. An *in vitro* GMPase activity assay confirms the regulatory role of the C-terminal left-handed β-helix domain, and structural analyses suggest the models of VTC1 hetero-complex with its interacting proteins. The structural information advances our insights into the different mechanisms involved in VTC1 regulation.

## Introduction

**FIGURE 1:**
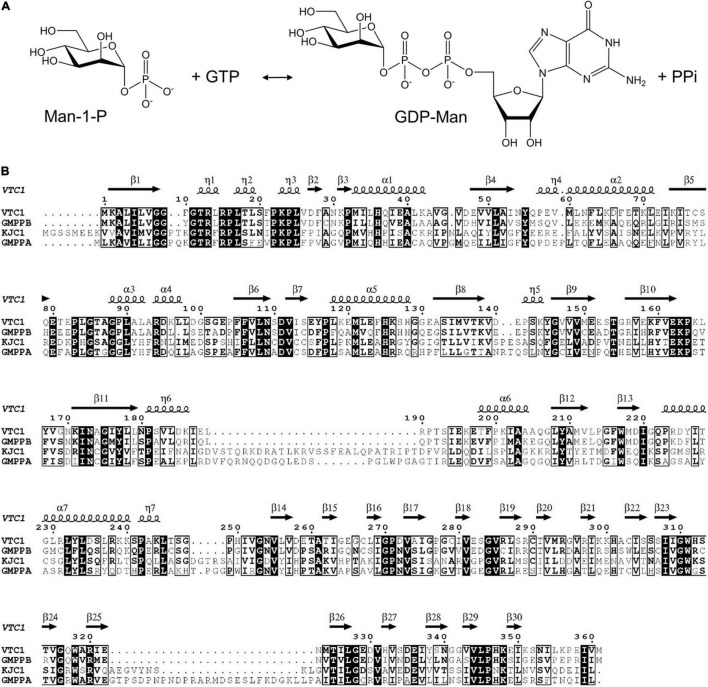
GMPase-catalyzed reaction and amino acid sequences of VTC1, GMPPB, KJC1, and GMPPA. **(A)** Interconversion of Man-1-P and GDP-Man. **(B)** Sequence alignment of VTC1, GMPPB, KJC1, and GMPPA. Conserved residues are in black background and similar residues are boxed. The secondary structural elements of VTC1 defined by DSSP are shown on the top.

Ascorbic acid (vitamin C) is essential for growth and development of animals and plants by playing key functions such as antioxidant and enzymatic cofactor (Englard and Seifter, 1986; Foyer et al., 2020). Plant vitamin C biosynthetic pathway contains conversion of D-mannose-1-phosphate (Man-1-P) to GDP-D-mannose (GDP-Man) (Figure 1A), a step catalyzed by the Man-1-P guanylyltransferase which is commonly named GDP-Man pyrophosphorylase (GMPase) (Wheeler et al., 1998; Smirnoff et al., 2001; Fenech et al., 2019). The first plant GMPase gene *VITAMIN C DEFECTIVE 1* (*VTC1*) was genetically defined in the model plant *Arabidopsis thaliana* (Arabidopsis) by screening the vitamin C-deficient mutants (Conklin et al., 1997, 1999), and was found to be identical to the gene *CYTOKINESIS DEFECTIVE1* (Lukowitz et al., 2001). Studies of the Arabidopsis *vtc1* mutants have revealed that *VTC1* is implicated in physiological processes including cell-wall formation (Nickle and Meinke, 1998; Nishigaki et al., 2021), cytokinesis (Falbel et al., 2003), control of the transcription of defense-related and senescence-associated genes (Pastori et al., 2003; Barth et al., 2004; Pavet et al., 2005), and ammonium sensitivity (Qin et al., 2008; Barth et al., 2010). These findings reflect the diverse functions of the nucleotide sugar GDP-Man in plants (Bar-Peled and O’Neill, 2011; Figueroa et al., 2021). At the protein level, VTC1 can interact with the COP9 signalosome subunit 5B (CSN5B) in response to light and darkness (Wang et al., 2013), and its CSN5B-interacting region is within the N-terminal 40-residue fragment (Li et al., 2016). It is expected that structural characterization of VTC1 will help to uncover the molecular basis for plant GMPase catalysis and regulation.

Structures of two GMPases have been reported, which are the crystal structure of GMPase from the hyperthermophilic bacterium *Thermotoga maritima* (TmGMP) (Pelissier et al., 2010) and the cryo-EM structure of human GMPase (GMPPA-GMPPB complex) that is arranged into a dodecamer (Zheng et al., 2021). These structures show that an oligomer-forming monomer (protomer) of GMPase consists of two domains: an N-terminal Rossmann fold-like domain and a C-terminal left-handed β-helix (LβH) domain. The Rossmann fold-like domain consists of a β-sheet core flanked mainly by α-helices and belongs to the glycosyltransferase (GT)-A fold according to the CAZy database (Coutinho et al., 2003; Drula et al., 2022). As this domain hosts the active site (Lairson et al., 2008), it is referred to as the catalytic domain. The LβH domain was first discovered in *Escherichia coli* UDP-N-acetylglucosamine acyltransferase (Raetz and Roderick, 1995) and was also found in potato ADP-glucose pyrophosphorylase (AGPase) (Jin et al., 2005). In plant AGPase, the LβH domain is involved in oligomerization and allosteric regulation of the AGPase activity (Figueroa et al., 2022). The LβH domain in TmGMP is responsible for dimerization (Pelissier et al., 2010). In human GMPase, the catalytically inactive GMPPA subunit and active GMPPB subunit assemble through three types of dimeric interface at which the LβH domain provides the main or sole inter-subunit contact (Zheng et al., 2021). Arabidopsis VTC1 shares 38%/62% amino acid sequence identity with human GMPPA/GMPPB, indicating that VTC1 and GMPPB are functional homologs (Figure 1B). However, unlike human GMPPB that forms a homo-dimer and coexists with GMPPA, VTC1 appears not to dimerize as revealed by yeast two-hybrid assay (Sawake et al., 2015). The GMPase activity of VTC1 can be upregulated by the GMPPA homolog KONJAC1 (KJC1) or KJC2 (Sawake et al., 2015). Thus, despite the amino acid sequence similarities among Arabidopsis and human GMPases, their function and underlying mechanism should differ from each other at certain aspects.

To uncover the structural basis of Arabidopsis GMPase catalysis, we determined the crystal structures of VTC1 in the unliganded and product-bound states at resolutions of 2.8 and 3.0 Å, respectively. VTC1 dimerizes *via* its LβH domain and forms a dodecamer in crystal. Structure of the GDP-Man-bound protomer delineates details of the active site. The regulatory role of LβH domain is confirmed by GMPase activity assay and the regulatory mechanism is suggested by structural comparison.

## Materials and Methods

### Protein Production and Crystallization

Production and crystallization of VTC1 were described previously (Zhao and Liu, 2016). The VTC1ΔC truncation mutant was generated with the Fast Mutagenesis System kit (TransGen Biotech, Beijing, China), and its expression and purification procedure was the same as that of the full-length VTC1.

### Structure Determination and Analysis

Diffraction data (Table 1) were collected at beamline BL17U (current BL02U1) at the Shanghai Synchrotron Radiation Facility (Wang et al., 2018) and were processed with HKL-3000 (Minor et al., 2006). The initial phase of the unliganded VTC1 was determined with Phaser (McCoy et al., 2007) using the cryo-EM structure of GMPPB (chain G of PDB entry 7D72) (Zheng et al., 2021) as search template. The model was rebuilt with AutoBuild (Terwilliger et al., 2008) and was refined with phenix.refine (Afonine et al., 2012) and Coot (Emsley and Cowtan, 2004) using the *Fo*–*Fc* and 2*Fo*–*Fc* maps. Model quality was evaluated with MolProbity (Williams et al., 2018). The product-bound VTC1 structure was determined using the unliganded VTC1 structure as search template. The structural figures were rendered with PyMOL (Schrödinger, LLC, New York). Sequence alignment was performed with Clustal Omega (Sievers et al., 2011) and drawn with ESPript (Robert and Gouet, 2014).

**TABLE 1:** Data collection and refinement statistics.

Crystal	Unliganded VTC1	Product-bound VTC1
PDB code	7 X 8J	7 X 8K
**Data collection**		
Space group	P2_1_	R32
Wavelength	0.979	0.979
Resolution range (Å)	50.00–2.80 (2.90–2.80)	50.00–3.00 (3.11–3.00)
Unit cell dimensions		
a, b, c (Å)	44.5, 146.9, 70.9	185.1, 185.1, 371.8
α, β, γ (°)	90, 100.5, 90	90, 90, 120
No. of measured reflections	82,303 (8,227)	545,568 (53,383)
No. of unique reflections	21,924 (2,165)	49,084 (4,853)
Redundancy	3.8 (3.8)	11.1 (11.0)
Completeness (%)	99.6 (99.7)	99.8 (100)
I/σI	16.1 (2.3)	26.0 (2.1)
R_*merge*_*^a^*	0.078 (0.523)	0.091 (1.0)
R_*pim*_*^b^*	0.047 (0.310)	0.031 (0.366)
*CC*_1/2_	0.967 (0.881)	0.969 (0.859)
Wilson B-factor	63.82	54.68
**Refinement statistics**		
Resolution range (Å)	31.48–2.798 (2.898–2.798)	30.82–3.002 (3.109–3.002)
R_*work*_*^c^*/R_*free*_*^d^*	0.215/0.258	0.234/0.260
No. of Molecules	2	4
No. of non-hydrogen atoms	5,546	11,191
Protein	5,430	11,002
Ligand	85	178
Water	31	11
Average B value (Å^2^)	63.81	58.65
Protein	63.69	58.41
Ligand	73.80	74.66
Water	57.48	41.78
R.m.s deviations		
Bond lengths (Å)	0.003	0.002
Bond angles (°)	0.56	0.61
Ramachandran plot		
Most favored (%)	95.77	95.03
Additional allowed (%)	0.56	4.69
Outlier (%)	0.00	0.28

*Values in parentheses are for the highest resolution shell.*

*^a^R_merge_ = Σ_hkl_Σ_i_| I_i_(hkl) – < I(hkl) > | /Σ_hkl_Σ_i_I_i_(hkl), where I_i_(hkl) is the ith observation of reflection hkl and < I(hkl) > is the weighted intensity for all observations i of reflection hkl.*

*^b^R_pim_ = R_merge_[1/(N–1)]^1/2^.*

*^c^R_work_ = Σ| | F_o_| –| Fc| | /Σ| F_o_|, where F_o_ and F_c_ are the observed and calculated structure factors, respectively.*

*^d^R_free_ is the cross-validated R factor computed for a test set of 5% of the reflections, which were omitted during refinement.*

### GDP-D-Mannose Pyrophosphorylase Activity Assay

The GMPase activity was measured based on previously reported procedure (Wu et al., 2002; Pelissier et al., 2010; Zheng et al., 2021). Assays were performed at 37^°^C with BioTek Synergy H1 plate reader (Agilent, Beijing, China) using a total volume of 100 μL. The reaction mixture contained 50 mM Tris-HCl, pH 7.5, 1 mM MgCl_2_, 1 mM DTT, 0.2 mM Man-1-P, and 0.2 mM GTP. Reaction was started by adding VTC1 (0.2 μM final concentration), lasted for 90 s, and stopped by boiling for 10 min. The product pyrophosphate (PPi) was converted to inorganic phosphate by Sigma-Aldrich pyrophosphatase (0.5 U/mL final concentration) at 25^°^C for 10 min. The content of inorganic phosphate was measured by the malachite green phosphate assay kit (Sigma-Aldrich, Shanghai, China) by recording the absorbance at 620 nm.

## Results

### Unliganded Structure

**FIGURE 2:**
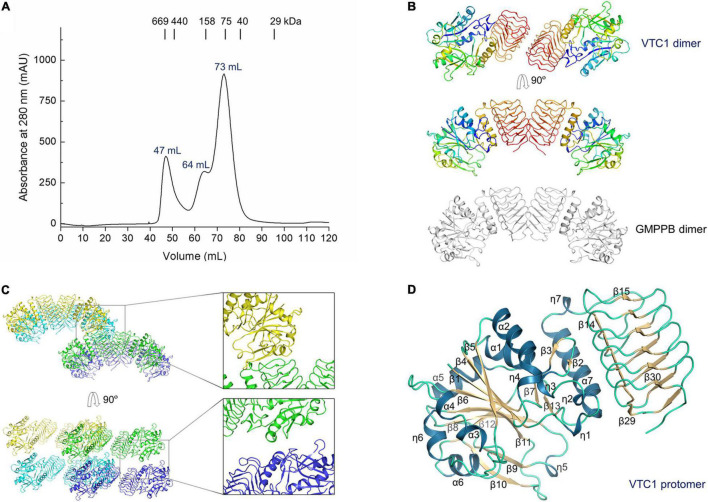
Purification of VTC1 and the unliganded structure. **(A)** SEC profile on a HiLoad 16/60 Superdex 200 column (GE Healthcare). The peak volumes of the VTC1 aggregate, tetramer, and dimer are labeled; the peak positions of the protein standards are indicated with corresponding molecular weights. **(B)** Ribbon representation of VTC1 dimer and GMPPB dimer (gray). Each VTC1 protomer is colored in rainbow with N-terminus in blue and C-terminus in red. **(C)** Crystal packing of VTC1. Four neighboring dimers are shown in green, blue, cyan, and yellow, respectively. The insets are enlarged view of the inter-dimer interfaces. **(D)** Ribbon representation of a VTC1 protomer. The secondary structures are colored as: dark blue, dark yellow and pale green for α-/η-helices, β-strands and loops. For clarity, only the first two and last two β-strands in the LβH domain are labeled.

The size exclusion chromatography (SEC) elution profile of VTC1 showed three peaks, corresponding to the dimeric, di-dimeric, and higher-order oligomeric states (Figure 2A). Recombinant VTC1 had a molecular weight of 41 kDa, and the apparent molecular weights of ∼80 and 170 kDa were estimated for the major and shoulder peaks, respectively. Crystallization of the purified VTC1 was reported previously (Zhao and Liu, 2016). The 2.8-Å unliganded structure was solved by molecular replacement using the model based on the cryo-EM structure of the GMPPA-GMPPB complex (Zheng et al., 2021). In crystal, VTC1 dimerizes in a manner similar to that of GMPPB, with the dimerization interface being solely mediated by the C-terminal LβH domain (Figure 2B). We then inspected the crystal-packing mode to obtain possible insight into how VTC1 oligomerizes. Two inter-dimer interfaces (Figure 2C) were found: the first interface is between catalytic domain and LβH domain, and the second interface results from side-by-side arrangement of two catalytic domains. The first interface could be of biological relevance while the inter-catalytic domain association may be merely due to crystal packing.

For structural description, one VTC1 protomer (chain A) was analyzed by the dictionary of secondary structure of proteins (DSSP) algorithm which defined the secondary structural elements (Kabsch and Sander, 1983). The VTC1 protomer has 7 α-helices, 7 η-helices, and 30 β-strands (Figures 1B, 2D). The catalytic domain comprises of 14 helical elements and 13 β-strands (β1–β13), and the LβH domain consists of 17 β-strands (β14–β30). The central β-sheet of the catalytic domain is made up by nine strands in the order of β5-β4-β1-β6-β11-β8-β12-β9-β10. Two small β-sheets (β2–β3 and β7–β13) lie near the C-terminal α-helix (α7) of the catalytic domain. The LβH domain has 5 three-β-stranded coils and one two-β-stranded coil.

### Overall Structure of the VITAMIN C DEFECTIVE 1-Product Complex

**FIGURE 3:**
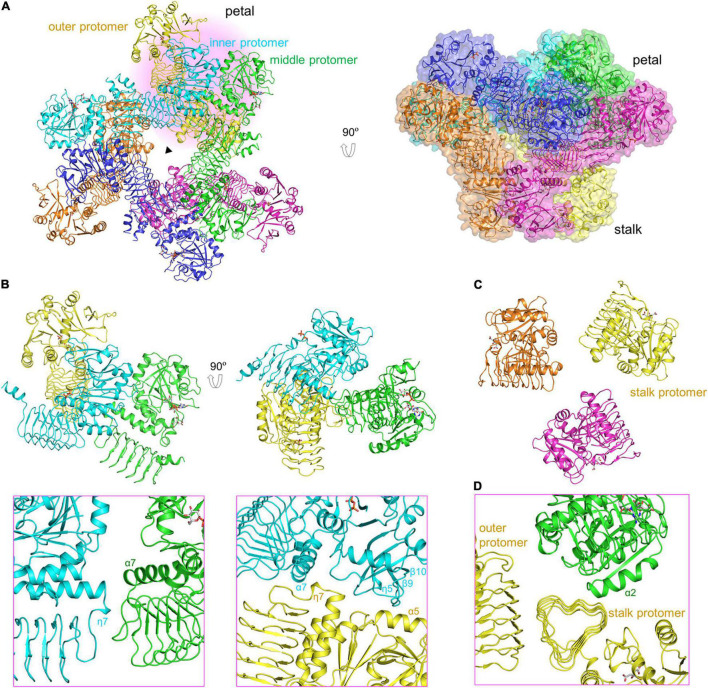
Dodecameric structure of product-bound VTC1. **(A)** Overall structure of dodecamer. Six dimers are colored in green, blue, cyan, yellow, brown, and magenta, respectively. GDP-Man, PPi and citrate are in stick representation. The three-fold symmetry center is denoted by a triangle. The petal on a light pink background is shown in **(B,D)**. A transparent surface of the dodecamer is shown in the right panel. **(B)** The inner, middle, and outer protomers. Upper left panel is the enlarged view of the petal. Two interfaces contributing to trimerization are enlarged and shown in the lower panels (pink boxes), where only two interacting protomers are shown for clarity and the orientations differ from those in upper panels to delimitate the interfaces. **(C)** Three stalk protomers. The image is the enlarged view of the stalk shown in the left panel in **(A)**. **(D)** The stalk-petal interface.

The 3.0-Å product-bound structure was solved based on the unliganded VTC1 structure. Different from the unliganded structure (Figure 2C), the overall structure of product-bound VTC1 has a threefold symmetry with each asymmetric unit having two dimers. Six dimers form a dodecameric assembly in shape of a three-petaled flower (Figure 3A). Each petal is composed of three protomers, which are referred to as the inner, middle, and outer protomers with respect to the symmetry center. The inner and middle protomers dimerize in the same way as the unliganded VTC1 dimer, and so do the outer protomer and the stalk protomer. Hence, the VTC1 dimerization interface is conserved irrespective of absence or presence of substrate/product. We then inspected the interfaces that contribute to trimerization of dimers, and found that each petal has two such interfaces (Figure 3B). The first one is between the N-terminal end (including η7) of inner protomer’s LβH domain and the N-terminal end of middle protomer’s LβH domain. The second interface involves both catalytic and LβH domains of the inner and outer protomers. The flower stalk is formed by three protomers not interacting with each other (Figure 3C). The stalk protomer, aside from dimerizing with the outer protomer, has an interface with the catalytic domain of the middle protomer *via* its LβH domain (Figure 3D). These three afore-mentioned interfaces bring the VTC1 dimers together.

### Active Site

**FIGURE 4:**
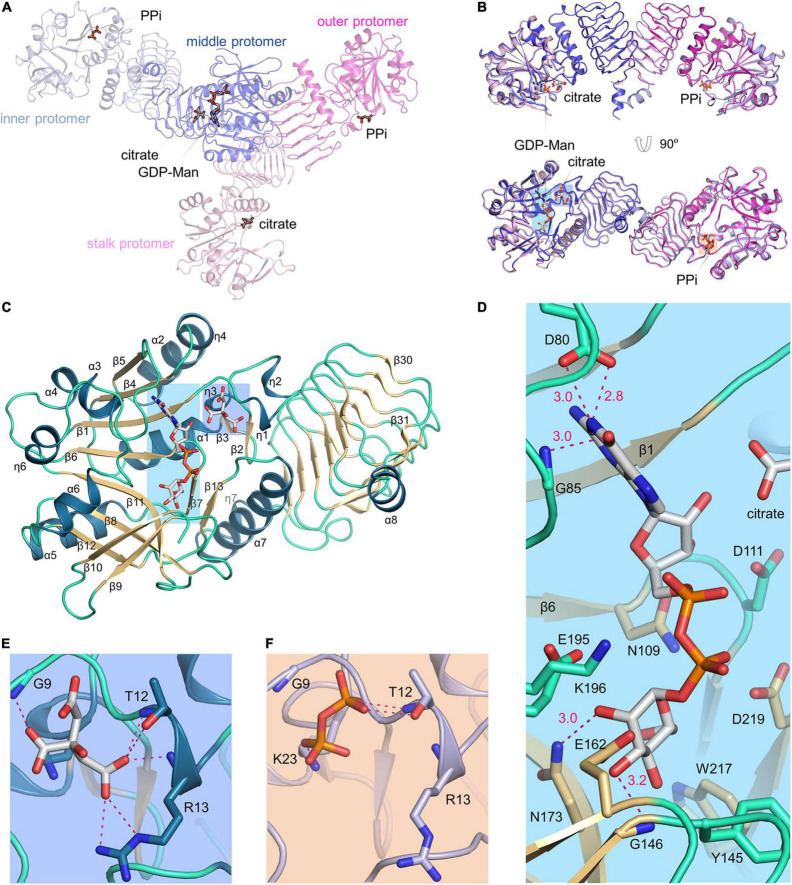
VTC1 active site. **(A)** Overall structure of the two dimers in an asymmetric unit. Proteins are in transparent ribbon representation and each protomer is shown in a different color. Ligands are in stick representation. **(B)** Dimer-to-dimer superimposition. Regions in colored background are shown in **(D–F)**. **(C)** Structure of the middle protomer. Color scheme is same as in Figure 2D. The middle protomer has an extra β-strand (β31) and a helix (α8) at the C-terminal end. The loop between β10 and β11 has a missing residue (Lys165). **(D)** VTC1–GDP-Man interactions. Side chains of Asp80, Asn109, Asp111, Tyr145, Glu162, Asn173, Glu195, Lys196, Trp217, and Asp219, and backbone amides of Gly85 and Gly146 are shown in sticks. Dashed magenta dots represent intermolecular hydrogen bonds with distance (Å) labeled. **(E)** VTC1–citrate interactions. **(F)** VTC1–PPi interactions. The inner protomer is shown and colored as in **(A)**.

The two dimers within an asymmetric unit bind a total of one GDP-Man, two inorganic PPi, and two citrates (Figure 4A). Specifically, the stalk protomer binds a citrate, the middle protomer binds a GDP-Man and a citrate, and the inner and outer protomers each bind a PPi. The two dimers can be aligned with a root mean square deviation of 0.49 Å. Their superimposition demonstrates that citrate and PPi bind to the same site on catalytic domain (Figure 4B). As the structures of four protomers are highly similar to each other, we used the middle protomer structure to describe the active site. The product GDP-Man binds at the edge of the central β-sheet (Figure 4C). The oxygen at guanine C6 forms a hydrogen bond with the backbone amide of Gly85, the guanine N1 forms two hydrogen bonds with the side chain of Asp80, and the hydroxyl groups at C2 and C3 of D-mannose form hydrogen bonds with the side chain amide of Asn173 and the backbone amide of Gly146, respectively (Figure 4D). The pyrophosphate moiety of GDP-Man is flanked by, but not directly interacting with the catalytically critical residues Glu195-Lys196, Asp111, and Asp219. The citrate is located near the loop between β1 and η1 (Figure 4E). It makes direct polar contacts with the backbone amide groups of Gly9, Thr12, and Arg13, the hydroxyl group of Thr12, and the guanidino group of Arg13. PPi binds to the same site as citrate (Figure 4F), but in the inner protomer, the interacting residues differ from the afore-mentioned residues in that Gly9 is not directly involved and Lys23 replaces Arg13 for ligand recognition.

### Protomer Conformations and GDP-D-Mannose Pyrophosphorylase Activity

**FIGURE 5:**
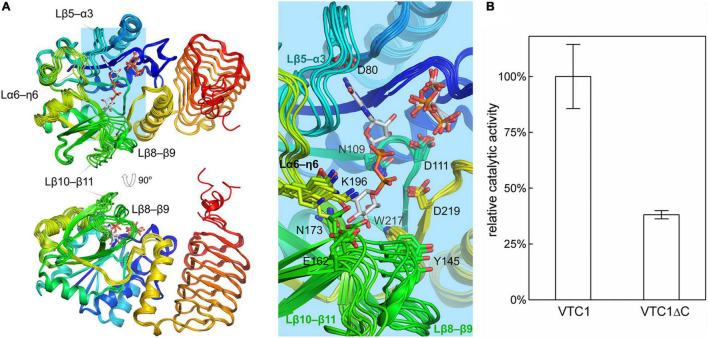
Superimposition of VTC1 protomers and activity assay. **(A)** Conformational ensemble containing six conformers. GDP-Man, PPi, and citrate are in sticks. VTC1 is colored in rainbow with N-terminus in blue and C-terminus in red. Inset: details of the active site. Side chains of residues shown in Figure 4D are drawn as lines. **(B)** GMPase activity of VTC1 and VTC1ΔC. Bars represent standard deviation from three independent experiments.

The unliganded and product-bound VTC1 structures provide an ensemble containing six conformers. Their superimposition reveals that the central β-sheet of the catalytic domain and coils of the LβH domain are conformationally conserved while variations occur in the loop regions (Figure 5A). Aside from the C-terminal end, four loops contributing to GDP-Man binding exhibit larger differences. These loops are connecting β5–α3, β8–β9, β10–β11, and η6–α6, respectively. The β5–α3 loop hosts the guanine-interacting residue Gly85; the β8–β9 and β10–β11 loops are near the mannose moiety; the η6–α6 loop contains Glu195-Lys196, which should participate in catalysis by interacting with the phosphate moiety of Man-1-P (Pelissier et al., 2010). In contrast to the four GDP-Man-binding loops, the β1–η1 loop contributing to citrate/PPi binding only undergoes small variations. The ensemble reflects that the LβH domain is dispensable for catalysis. We then measured the GMPase activity of VTC1 and its truncation mutant (VTC1ΔC) that lacks the C-terminal 139 residues. Compared with the full-length VTC1, VTC1ΔC retained, but only ca. one third of the GMPase activity (Figure 5B). This result indicates that the C-terminal region of VTC1 is involved in the positive regulation of catalytic domain by forming a homo-oligomer.

## Discussion

**FIGURE 6:**
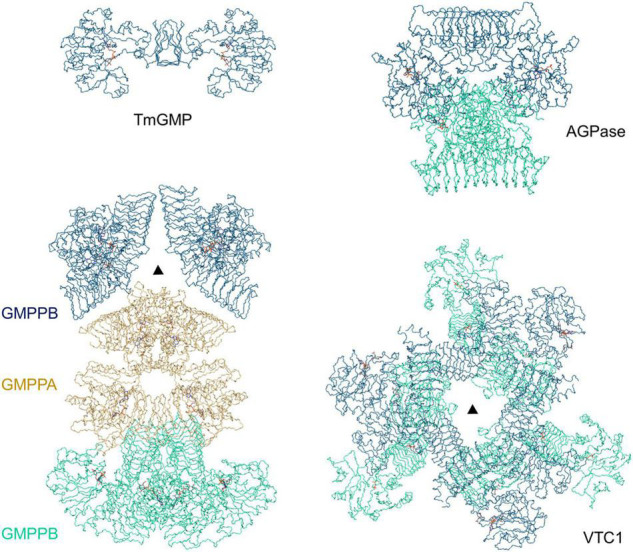
Comparison of TmGMP, GMPPA-GMPPB, AGPase, and VTC1. The corresponding PDB entries are 2 × 5Z, 7D72, 1YP4, and 7 × 8K. The half GMPPA-GMPPB complex in a pseudo threefold symmetry is shown for comparison with the VTC1 dodecamer. TmGMP, two GMPPB dimers, two AGPase dimers, and three VTC1 dimers are shown in dark blue; GMPPA (di-dimer) is shown in dark yellow; two GMPPB dimers, two AGPase dimers, and three VTC1 dimers are in pale green. Triangle denotes the threefold symmetry center.

Our VTC1 structures revealed previously uncharacterized features of GMPase. Structural comparison of TmGMP, GMPPA-GMPPB, and AGPase with VTC1 shows that their oligomers vary with size and organization (Figure 6). TmGMP has a small LβH domain with only two and a half coils, and dimerizes by pairing of the extra β-strand (Pelissier et al., 2010). The GMPPA-GMPPB dodecamer can be divided into two hetero-hexamer, each being composed of two GMPPA-GMPPB hetero-dimers and a GMPPB homo-dimer (Zheng et al., 2021). GMPPA and GMPPB dimerize in a same way as the GMPPB homo-dimer. The two hetero-dimers and one GMPPB homo-dimer are arranged in a pseudo threefold symmetry. The potato AGPase exists as a homo-tetramer which can be considered as a dimer of dimers (Jin et al., 2005). The AGPase LβH domain mediates dimerization in a way as its TmGMP counterpart, and the catalytic domain is solely responsible for dimerization of dimers. The VTC1 dodecamer consists of a top hexamer and a basal hexamer. Trimerization of dimers forms the top hexamer, while the basal hexamer can be viewed as an appendant of the top hexamer because there is no direct inter-dimer interaction within the basal hexamer (Figure 3). The top hexamer differs from the hetero-hexamer of GMPPA-GMPPB in that the inter-dimer LβH domains are arranged in a partial pairing way, and six LβH domains are arrayed as a triangle.

The structures provide clues for explaining previous experimental data. Yeast two-hybrid assay showed that VTC1 could form a hetero-complex with KJC1 but not a homo-complex with itself (Sawake et al., 2015). The undetected VTC1 homo-complex could be due to method limitation, since VTC1 can form homo-complex at different oligomeric states as described in this work. The KJC1-VTC1 hetero-complex could be either similar to the GMPPA-GMPPB complex or the VTC1 homo-complex. Compared with GMPPA, KJC1 has a 7-residue longer loop between η6 and α6 and a 19-residue shorter insertion (β25–β26) of the LβH domain (Figure 1B). Both loops have been found to be involved in allosteric regulation of GMPPA-GMPPB (Zheng et al., 2021). While it remains unclear how KJC1 regulates the GMPase activity of VTC1 until the hetero-complex structure is available, the differences between GMPPA and KJC1 for these two loops suggest that they may act in different ways. In addition, VTC1 was found to directly interact through its N-terminal region with the metalloprotease catalytic center CSN5B (Wang et al., 2013; Jin et al., 2014). The VTC1 structures indicate that the region should be between β1 and α1 for it is exposed and that the CSN5B-interacting residue Asp27 is within this region (Li et al., 2016).

The structural ensemble of VTC1 protomers also reflects the dynamics during catalytic process (Figures 4, 5). The β5–α3 loop near guanine-binding site, the critical η6–α6 loop, and the β8–β9 and β10–β11 loops around mannose-binding site undergo large conformational changes. The substrate GTP should bind to the active site with its guanine moiety in the same orientation as in the product GDP-Man, and with its β- and γ-phosphate moieties pointing toward the β1–α1 loop. The Man-1-P-binding mode is expected to be the same with the moiety of itself in GDP-Man. Upon reaction, the product PPi should be repulsed from the pyrophosphate moiety and be accommodated in the β1–η1 loop.

Taken together, our study has uncovered the molecular basis for VTC1 oligomerization and delineated the active site. The structural information lays a foundation for future work on plant GMPases which undergo an intricate mechanism of assembly and hence a fine regulation due to the diverse function of GDP-Man.

## Data Availability Statement

The datasets presented in this study can be found in online repositories. The names of the repository/repositories and accession number(s) can be found below: https://doi.org/10.2210/pdb7X8J/pdb; https://doi.org/10.2210/pdb7X8K/pdb.

## Author Contributions

CZ, SZ, and Y-SL performed the experiments. CH, XW, and LL analyzed the structure. SZ and LL designed the study. CZ and LL wrote the manuscript. All authors contributed to the article and approved the submitted version.

## Conflict of Interest

The authors declare that the research was conducted in the absence of any commercial or financial relationships that could be construed as a potential conflict of interest.

## Publisher’s Note

All claims expressed in this article are solely those of the authors and do not necessarily represent those of their affiliated organizations, or those of the publisher, the editors and the reviewers. Any product that may be evaluated in this article, or claim that may be made by its manufacturer, is not guaranteed or endorsed by the publisher.
